# Immune Quiescence: a model of protection against HIV infection

**DOI:** 10.1186/1742-4690-10-141

**Published:** 2013-11-20

**Authors:** Catherine M Card, Terry Blake Ball, Keith R Fowke

**Affiliations:** 1 Department of Medical Microbiology, University of Manitoba, 539-745 Bannatyne Ave., Winnipeg R3E 0 J9, Canada; 2 Department of Immunology, University of Manitoba, Winnipeg, Canada; 3 Department of Medical Microbiology, University of Nairobi, Nairobi, Kenya; 4 National Microbiology Laboratories, Public Health Agency of Canada, Winnipeg, Canada; 5 Department of Community Health Sciences, University of Manitoba, Winnipeg, Canada; 6 Current affiliation: Institute for Bioengineering, École Polytechnique Fédérale de Lausanne (EPFL), Lausanne, Switzerland

**Keywords:** HIV, Immune activation, Immune quiescence, HIV resistance, HIV susceptibility, HESN

## Abstract

Aberrant immune activation is a strong correlate of HIV disease progression, but little is known about how immune activation alters susceptibility to HIV infection. Susceptibility to HIV infection varies between individuals, but the immunological determinants of HIV transmission are not well understood. Here, we present evidence from studies of HIV transmission in the context of clinical trials and HIV-exposed seronegative (HESN) cohorts that implicates elevated immune activation as a risk factor for acquiring HIV. We propose a model of protection from infection based on a phenotype of low baseline immune activation referred to as *immune quiescence*. Immune quiescence is evidenced by reduced expression of T cell activation markers, low levels of generalized gene transcription and low levels of proinflammatory cytokine and chemokine production in the periphery and genital mucosa of HESN. Since HIV preferentially replicates in activated CD4+ T cells, immune quiescence may protect against infection by limiting HIV target cell availability. Although the determinants of immune quiescence are unclear, several potential factors have been identified that may be involved in driving this phenotype. HESN were shown to have elevated proportions of regulatory T cells (Tregs), which are known to suppress T cell activation. Likewise, proteins involved in controlling inflammation in the genital tract have been found to be elevated in HESN. Furthermore, expression of interferon regulatory factor 1 (IRF-1) is reduced in HESN as a consequence of genetic polymorphisms and differential epigenetic regulation. Since IRF-1 is an important regulator of immune responses, it may play a role in maintaining immune quiescence. Based on this model, we propose a novel avenue for HIV prevention targeted based on reducing host mucosal immune activation.

## Introduction

Despite the advances in the management of HIV infection, new HIV infections remain high. A combination of proven prevention approaches and novel approaches are needed to stem this tide. While it is known that immune activation drives HIV disease progression, its role in HIV susceptibility is unclear. In this review, we will present evidence that immune activation is a key determinant in viral transmission and propose a model of immune quiescence (IQ) as a mechanism of protection from infection. Reducing susceptibility to HIV infection by inducing IQ represents a novel mechanism of HIV prevention.

## Review

### HIV dependency on immune activation

HIV replication and immune activation are critically intertwined. Immune activation has been recognized as a significant factor in the destruction of the immune response and rapid HIV disease progression [1], but the relationship between immune activation and susceptibility to HIV infection is not well defined. *In vitro* studies demonstrated that quiescent CD4+ T cells can be infected by HIV, but viral replication is inefficient [2]. As such, HIV preferentially establishes productive infection in activated T cells [3,4]. One reason for this preference is the large number of host factors required for efficient HIV replication [5-7], which are primarily expressed in activated cells. Based on these observations, it is reasonable to hypothesize that individuals with lower levels of immune activation would have lower susceptibility to HIV infection. Indeed, in the Pumwani HESN cohort, reduced susceptibility of unstimulated PBMC to *in vitro* HIV infection was linked to low levels of activated CD4+ CD69+ T cells and elevated levels of immunosuppressive Tregs [8], in agreement with earlier data showing elevated HIV susceptibility of lymphocytes from individuals with high immune activation [9]. These data support a previous study, which showed that unstimulated cells from HESN had reduced susceptibility to infection compared to controls, but levels of infection were comparable when cells were pre-activated with a mitogen [10]. In addition to these *in vitro* data, evidence gathered from various models support this immune quiescence hypothesis, as detailed in the sections below.

### Models informing the role of immune activation in HIV transmission

#### Microbicide trials

Functional studies conducted in conjunction with clinical trials of microbicide candidates have been instrumental in studying the determinants of HIV transmission. Early microbicide candidates were not effective at preventing acquisition, and some were unfortunately associated with increased HIV transmission rates. For instance, the microbicides cellulose sulfate and nonoxynol-9 disrupted tight junctions between epithelial cells and caused inflammation in the female genital tract (FGT), characterized by enhanced IL-1α and IL-8 expression and activation of the NF-kB pathway [11-13]. It has been suggested that this inflammation resulted in more HIV target cells at the site of viral exposure, thereby increasing the risk of HIV infection [13]. The most promising microbicide candidate to date was a 1% tenofovir gel, which demonstrated a 39% protective effect when tested in the CAPRISA 004 trial [14]. In that trial, the extent of immune activation prior to exposure was correlated directly with increased risk of HIV seroconversion, irrespective of microbicide or placebo use [15]. Systemic immune activation was marked by activation of natural killer (NK) cells, elevated levels of plasma cytokines and elevated levels of CD8+ T cell degranulation relative to women who remained HIV negative [15]. The authors of this study concluded that suppression of innate immune activation should be considered when developing the next generation of antiretroviral microbicides [15].

#### HIV-Exposed Seronegative (HESN)

It is appreciated that not everyone who is exposed to HIV will necessarily become infected. Individuals demonstrating natural resistance to HIV infection, referred to as HIV-exposed seronegative (HESN), are considered to be a model on which novel prevention efforts can be based. We have followed a cohort of commercial sex workers (CSW) from the Pumwani district of Nairobi, Kenya since 1984. A proportion of CSW in the Pumwani cohort (~5-10%) have been identified as HESN [16]. Several correlates of protection have been identified in various HESN cohorts, but no single factor accounts for all cases of resistance to infection. However, emerging evidence from studies of the Pumwani cohort and others, implicates immune quiescence in protection against infection. Immune quiescence refers to a state of low baseline immune activation, which we propose protects against infection by limiting HIV target cells and substrates available for HIV replication.

### T cell immune quiescence

To evaluate the role of T cell activation on resistance to infection, T cells were phenotyped in HESN and HIV-negative, yet susceptible, CSW from the Pumwani cohort. Reduced CD69+ CD4+ and CD8+ T cells were observed in HESN when compared with controls, indicating low levels of T cell activation [17].

Further evidence of low T cell activation in HESN participants of the Pumwani cohort is provided by a study in which microarray technology was implemented to investigate CD4+ T cell function [18]. These analyses revealed that unstimulated CD4+ T cells from HESN had a lower level of gene expression than high risk HIV-negative controls, suggesting a quiescent cellular state. A subsequent study confirmed these observations by examining gene expression in whole blood [19]. Genes involved in T cell receptor signaling and host factors required for HIV replication were among the underexpressed genes in HESN [18,19]. Unstimulated cells from HESN were also found to secrete lower levels of cytokines *ex vivo* compared to control groups, but this difference in cytokine secretion was not observed following stimulation [18], indicating that HESN have a low baseline level of cellular activation, but respond normally to stimulation and are not immunosuppressed. Likewise, a recent study demonstrated reduced production of proinflammatory cytokines and chemokines by unstimulated lymphoctyes from HESN relative to low-risk HIV-negative controls [20]. While the majority of cytokines were comparable between groups following stimulation of peripheral blood mononuclear cells (PBMC), IL-17 and IL-22 remained lower in HESN compared to controls, suggesting blunted Th17 responses in HESN [20].

Evidence for T cell immune quiescence in HESN is not limited to the Pumwani cohort. For instance, low frequencies of CD4+ and CD8+ T cells expressing the activation markers HLA-DR, CD38, CD70 and Ki67 were identified in HESN men who have sex with men (MSM) [21], and studies of serodiscordant couples found reduced expression of CD38 [22], HLA DR and CCR5 [10] on CD4+ T cells from the uninfected partner. Lymphocytes isolated from HESN CSW from a cohort in Côte d’Ivoire expressed lower levels of CD69 and reduced proinflammatory cytokines and chemokines following allostimulation compared to low-risk HIV-negative controls [23]. Other HESN studies of CSW from the Central African Republic and intravenous drug users in Vietnam showed that despite higher CD8+ T cell activation, there were significantly fewer CCR5+ CD4+ T cells in the blood of HESN [10,24]. Also, independent studies of uninfected hemophiliacs who were exposed to HIV-contaminated blood products [25] and of high-risk MSM [21] found reduced lymphoproliferation in HESN compared to healthy controls. Together, these studies from a variety of cohorts showed a common phenotype of reduced systemic immune activation in HESN.

### Immune quiescence in the female genital tract

The majority of HIV transmissions occur at the genital mucosa. As such, mucosal immunology is a central issue in studies of HIV susceptibility. Evaluations of inflammation in the female genital tract have demonstrated immune quiescence at the mucosal level. For instance, cervical mononuclear cells (CMC) from HESN from the Pumwani cohort were shown to have reduced expression of genes encoding proinflammatory cytokines compared to high-risk HIV-negative CSW from the same cohort [20]. Of note, reduced cervical expression of IL-17 and IL-22 were observed in that study, consistent with observations from the peripheral blood. Th17 cells expressing these cytokines may be an important target for HIV infection, as these cells are enriched in the CMC population and are preferentially depleted in HIV-infected individuals [26].

CMCs from HESN from the Pumwani cohort were also observed to have reduced expression of genes encoding various pattern recognition receptors (PRRs), including toll-like receptor (TLR)-2, TLR-4, TLR-7, TLR-8, RIG-I and MDA5. HESN also had reduced expression of UNC93B, which is involved in trafficking of TLRs within the cell. CMCs also produced low basal levels of cytokines in culture, but were highly responsive to stimulation with the TLR7/8 ligand ssRNA40, suggesting that despite low levels of PRRs at baseline, HESN are capable of mounting an appropriate anti-viral response [27].

Consistent with reduced proinflammatory cytokine expression by CMCs, reduced expression of IL-1α, IL-8, MIG and IP-10 were found in HESN cervicovaginal lavage (CVL) samples compared to high-risk HIV-negative controls [28]. MIG and IP-10 are both IFNγ-inducible chemokines that bind the receptor CXCR3, inducing recruitment of activated T cells. Lower levels of these chemokines would result in a decreased recruitment of activated T cells to the FGT. Consistent with this, a trend toward reduced proportions of cervical HLA DR + CD8+ T was observed in HESN [28].

Overall, these studies described HESN individuals with lower levels of inflammation at the FGT that may result in fewer HIV target cells at the point of first encounter with HIV.

### Drivers of immune quiescence

#### Regulatory T cells (Tregs)

Due to the capacity of Tregs to suppress cellular activation, the role of these cells in maintaining T cell quiescence was investigated in HESN from the Pumwani cohort. Tregs were found to be elevated in HESN relative to high-risk HIV-negative controls [17].

Tregs suppress T cell activation and responses through multiple mechanisms, both indirectly, through production of anti-inflammatory cytokines or interactions with dendritic cells (DC), and directly, though direct contact with CD4+ and CD8+ T cells [29]. As such, elevated levels of Tregs in HESN may protect against infection by limiting activation of conventional CD4+ T cells. Although Tregs are known to suppress antigen-specific T cell responses, one study found that PBMC from HESN with elevated Treg frequencies did not show enhancement of HIV-specific T cell proliferation upon Treg depletion [30], suggesting that HESN can maintain HIV-specific T cell responses in the presence of Tregs.

Tregs may also protect against infection by additional mechanisms. For example, Tregs can directly suppress HIV replication through the activity of cyclic adenosine monophosphate (cAMP), which negatively regulates HIV replication [31,32].

Paradoxically, Tregs can be infected with HIV [33-35], suggesting that elevated Tregs may actually provide additional target cells for HIV infection. However, although X4-tropic viruses preferentially target Tregs, these cells are less susceptible to infection with R5-tropic HIV strains than conventional CD4+ T cells [35]. Since mucosal HIV transmission occurs in a CCR5-dependent manner [36], Tregs may not be a major target cell during early transmission events. Furthermore, the Treg transcription factor Foxp3 has been shown to repress HIV long terminal repeat (LTR) transcription in infected cells [37,38], although one study presented the disparate finding that Foxp3 enhanced HIV LTR activity [39]. Therefore, elevated Treg levels may drive lower levels of immune activation in HESN subjects.

#### Antiproteases

In addition to regulatory cell subsets, other potential drivers of the immune quiescent phenotype include innate proteins that play a role in the control of inflammation. To identify putative factors involved in mediating resistance against infection, a proteomics approach was applied to screen for differences in CVL proteins between HESN and high-risk HIV-negative CSW from the Pumwani cohort. These comparisons revealed that HESN overexpressed several antiproteases, including members of the serpin A family, cystatin B, Elafin and A2ML1 [40,41]. In line with these observations, Elafin has been identified as a correlate of protection in HESN [42]. These antiproteases have anti-inflammatory activity and their presence in the mucosa may limit immune activation and HIV target cell availability. Antiproteases are usually induced in the presence of inflammatory cytokines and chemokines. However, in HESN, serpins remain high in the absence of inflammation and correlations between serpins and proinflammatory factors are absent [28], suggesting that constitutive expression of serpins may help to maintain low inflammation in the FGT of HESN CSW.

#### Interferon regulatory factor-1 (IRF-1)

IRF-1 is a key transcriptional activator and repressor involved in inducing expression of inflammatory genes in response to IFN signaling. In addition, IRF-1 can activate transcription of the HIV genome during early stages of HIV infection [43]. In the Pumwani cohort, specific polymorphisms in *IRF1* were shown to result in reduced IFNγ-stimulated IRF-1 protein expression and were associated with resistance to infection [44], but not altered disease progression [45], suggesting that the protective effect is limited to early events in HIV infection. HESN individuals who lacked the “protective” IRF-1 genotype were shown to have reduced IRF-1 response potential as a consequence of differential epigenetic regulation [46]. The kinetics of the IRF-1 response to IFNγ stimulation in HESN was transient and immediately silenced in HESN. This was in contrast to HIV-susceptible individuals, who demonstrated a sustained IRF-1 response. These data emphasize the observation that immune quiescence is not equivalent to immunosuppression, as HESN are able to mount robust immune responses at the innate [46] and adaptive [18] levels. However, these responses are regulated such that immune activation is quickly resolved, restoring a quiescent basal state.

Murine studies have demonstrated that IRF-1 negatively regulates Treg development by repression of the Treg transcription factor Foxp3 [47]. As mentioned above, Tregs are elevated in HESN, but the causes of this expansion are unknown. It is conceivable that reduced IRF-1 protein expression alleviates repression of Treg differentiation in HESN, allowing for higher Treg levels to develop, although further studies are needed to directly address the interplay between IRF-1 and Tregs. Along these same lines, the chemokines MIG and IP-10, which were shown to be reduced in the cervical mucosa of HESN [28], are upregulated by IFNγ stimulation, suggesting that reduced expression of IRF-1 may contribute to reductions in mucosal chemokines. Future studies should focus on clarifying the role of the IFNγ signaling pathway and IRF-1 in mediating the immune quiescent phenotype in HESN.

### Immune quiescence model of protection against HIV infection

**Figure 1:**
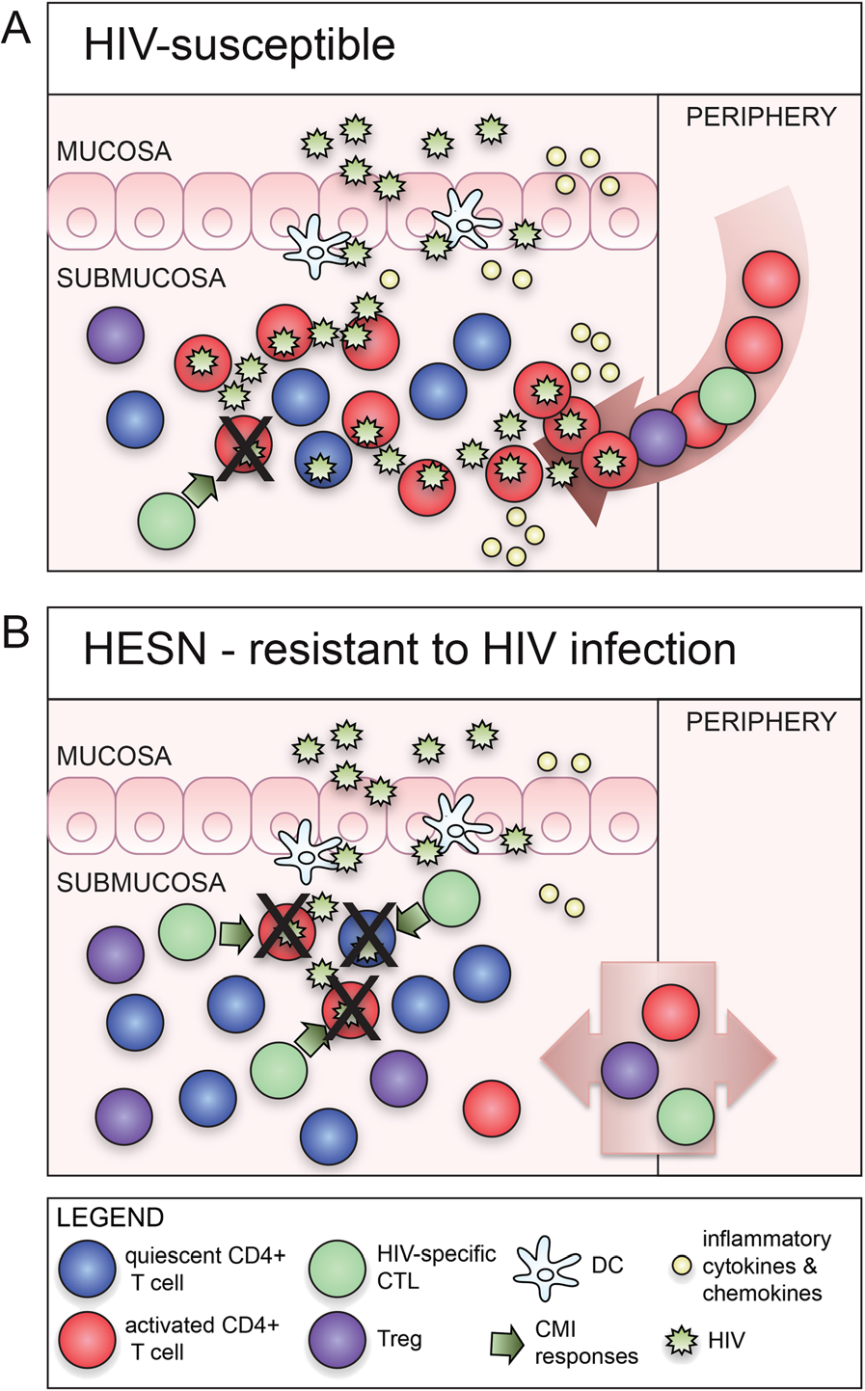
**Immune Quiescence Model of protection from HIV infection. (A)** In HIV-susceptible individuals, infection is established in resting and activated CD4+ target cells. Inflammation drives infiltration of activated target cells, which fuel viral propagation and dissemination of infection. **(B)** In HESN, elevated levels of Tregs maintain low levels of T cell activation, leading to reduced availability of activated CD4+ target cells. HIV establishes infection in resting T cells, which support low levels of virus replication. Reduced expression of proinflammatory cytokines and chemokines prevents infiltration of activated target cells. Productive infection is therefore reduced in HESN, allowing HIV-specific CTL sufficient opportunity to clear the low-level infection and prevent dissemination.

As described in the sections above, elevated levels of immune activation are associated with increased risk of HIV infection, and protection from infection correlates with immune quiescence. This is marked by reduced T cell activation, reduced gene transcription, reduced lymphoproliferation, reduced peripheral and mucosal cytokine and chemokine production, elevated Tregs, elevated mucosal anti-proteases and reduced IRF-1 expression and IFNγ-responsiveness. Previous HESN studies have demonstrated HIV-specific T cell responses, both systemically and mucosally [48]. Taken together, these observations can be used to frame a model of reduced susceptibility to infection. In HIV-susceptible individuals, HIV exposure leads to penetration of the mucosal barrier and establishment of infection in a small focus of CD4+ target cells. Inflammation resulting from infection drives infiltration of activated target cells, which fuel propagation of infection and dissemination to lymphoid tissues. Once dissemination occurs, the infection cannot be cleared (Figure 1A). In contrast, in HESN, innate molecules in the FGT mucosa may limit establishment of the initial focus of infection through direct anti-viral activity and maintenance of mucosal integrity. While it is likely that some viral particles penetrate the epithelial barriers and gain access to target cells in the submucosa, Tregs and antiproteases maintain low levels of T cell activation, leading to reduced availability of activated CD4+ target cells. Consequently, the target cell population is limited to resting T cells, which do not effectively support viral replication. Reduced expression of proinflammatory cytokines and chemokines prevents infiltration of activated target cells. As such, the focus of viral replication remains small and unproductive in HESN, allowing innate or adaptive cells, such as NK or HIV-specific CTL, opportunity to clear the low-level infection and prevent dissemination (Figure 1B).

### Support for immune quiescence from animal models

The immune quiescence model is supported by studies of mucosal simian immunodeficiency virus (SIV) transmission in non-human primates (NHP). In acute infection of rhesus macaques, a small founder population of infected cells is established in the submucosa within the first few days following viral challenge. This founder population is comprised of mainly resting CD4+ T cells, as these cells outnumber activated cells. Local expansion of the founder population occurs through low-level viral replication in resting cells. However, the infection is fuelled by an influx of activated target cells in the inflammatory infiltrate resulting from the innate response to SIV [49]. In this way, SIV exploits the inflammatory immune response to gain access to target cells for viral replication and dissemination of infection. Interestingly, topical administration of the anti-inflammatory compound glycerol monolaurate blunts the inflammatory response, preventing infiltration of activated target cells to the FGT, resulting in protection from SIV infection [50]. Furthermore, vaccination of macaques with a tolerogenic vaccine prior to SIV exposure resulted in development of CD8+ Tregs that suppressed CD4+ T cell activation and prevented the establishment of SIV infection [51].

In SIV-infected animals, SIV-specific CD8+ T cells are detectable by the second week of infection. However, infection is well established by this time, such that the ratio of HIV-specific CD8+ T cells to infected target cells is too low to prevent further viral replication and dissemination [52]. Nonetheless, mucosal HIV-specific T cells present at the time of inoculation have been shown to be protective against simian-human immunodeficiency virus (SHIV) challenge [53]. We propose that in HESN, mucosal HIV-specific T cells, which have been previously identified in HESN [54], are present early enough to clear the initial focus of infection prior to expansion and dissemination.

### Contradictory evidence

Data that conflict with the immune quiescence hypothesis appear in reports of elevated immune activation as a correlate of protection in HESN. Elevated levels of T cell activation and cytokine production have been found in systemic or mucosal samples from cohorts of HESN injection drug users (IDU) [24,55,56] and uninfected partners of HIV + patients [24,55-57]. However, in the Vietnamese HESN IDU cohort [24], activation of the CD8+ and NK T cell compartments occurred at the same time as reduced levels of CD4+ CCR5+ T cell targets. This suggests the possibility that in certain cohorts, CD4+ T cell quiescence can occur concomitantly with activation of other immune cell subsets. It is possible that other disparate findings may be reconciled by differences in the site or intensity of HIV exposure, presence of other immunogenic factors or differences in assays used to characterize immune activation.

### Translation of IQ into novel prevention approaches

An important consideration is the translation of the immune quiescence hypothesis into interventions that prevent HIV infection. Our model suggests that inducing immune quiescence at the site of HIV exposure will reduce the number of activated target cells, thereby preventing infection or limiting it to small foci of infected resting target cells, which can be cleared by mucosal HIV-specific T cells or innate mechanisms. A practical method of inducing immune quiescence in the mucosa may be a microbicide that incorporates anti-inflammatory mediators into the formulation, perhaps in combination with antiretroviral compounds that have shown moderate efficacy in recent trials [14]. This approach has been suggested based on results of functional studies performed in the wake of the CAPRISA 004 trial [15]. Suitable compounds would be drugs that are already approved by the FDA, have outstanding safety records, have a demonstrated ability to reduce excessive immune activation, and are affordable for those in developing countries. There are a number of potential candidate anti-inflammatory compounds, such as type I interferon blockers [58], cyclooxygenase type 2 inhibitors [59], statin inhibitors [60], chloroquine [61,62] and others, that address some or all of these criteria. An advantage of the approach of inducing the IQ phenotype is that it is targeting the host immune system and not the virus, thereby limiting any pressure on HIV to mutate to evade the mechanism.

In implementing induction of immune quiescence as a strategy to reduce HIV transmission, caution needs to be taken to ensure that the limitation of excessive mucosal T cell activation does not result in the induction of an inability to mount a mucosal immune response. Indeed, it may seem counter-intuitive to try to limit excessive immune activation on the one hand, while at the same time inducing anti-viral immune responses with an HIV vaccine. However, these goals are not incongruent. Limiting HIV target cells by inducing IQ in the FGT can be accomplished on its own, independent of an HIV vaccine. Should this strategy be used in conjunction with an HIV vaccine, it would be important to activate and educate the immune system with the vaccine first, prior to inducing IQ in the FGT. It is important to recall that the induction of IQ is not equivalent to the induction of anergy. As shown, individuals with high levels of quiescent T cells are capable of responding to recall antigen or mitogens [63]. Indeed, in the Pumwani HESN cohort we observe a quiescent phenotype [63] and HIV-specific immunity [64,65]. Therefore, HIV-specific immunity in the context of decreased generalized T cell immune quiescence is not only highly plausible, but it has been observed. As such, candidate formulations designed to induce IQ will need to be tested for the ability to reduce immune activation and inflammation without interfering with HIV-specific CD8+ T cell responses or innate antiviral mechanisms such as NK cells.

## Conclusions

There are multiple examples that show pre-existing immune activation prior to HIV exposure is a risk factor for HIV infection. Informed by the study of HESN cohorts, the IQ model suggests that reduced immune activation may provide protection against HIV infection by limiting the pool of activated target CD4+ T cells susceptible to HIV infection. Differences in Tregs, antiproteases and IRF-1 may drive the IQ phenotype. Future studies should focus on how to induce immune quiescence in combination with other strategies such as microbicides and HIV vaccines.

## Abbreviations

HIV: Human immunodeficiency virus; FGT: Female genital tract; NK: Natural killer; HESN: HIV-exposed seronegative; IQ: Immune quiescence; CSW: Commercial sex worker; PBMC: Peripheral blood mononuclear cell; MSM: Men who have sex with men; CMC: Cervical mononuclear cell; PRR: Pattern recognition receptor; TLR: Toll like receptor; CVL: Cervicovaginal lavage; DC: Dendritic cell; Treg: Regulatory T cell; cAMP: cyclic adenosine monophosphate; LTR: Long terminal repeat; IRF-1: Interferon regulatory factor 1; NHP: Non-human primate; SIV: Simian immunodeficiency virus; SHIV: Simian-human immunodeficiency virus; IDU: Injection drug user.

## Competing interests

The authors declare that they have no competing interests.

## Authors’ contributions

CMC, TBB and KRF conceived of the review topic. All authors contributed to the preparation of the manuscript. All authors read and approved the final manuscript.
